# Development of sequencing-based methodologies to distinguish viable from non-viable cells in a bovine milk matrix: A pilot study

**DOI:** 10.3389/fmicb.2022.1036643

**Published:** 2022-11-17

**Authors:** Min Yap, Orla O’Sullivan, Paul W. O’Toole, Paul D. Cotter

**Affiliations:** ^1^Teagasc Food Research Centre, Moorepark, Fermoy, Ireland; ^2^School of Microbiology, University College Cork, Cork, Ireland; ^3^APC Microbiome Ireland, Cork, Ireland

**Keywords:** next-generation sequencing, microbial viability, Illumina, Oxford nanopore, PMA, milk microbiome

## Abstract

Although high-throughput DNA sequencing-based methods have been of great value for determining the composition of microbial communities in various environments, there is the potential for inaccuracies arising from the sequencing of DNA from dead microorganisms. In this pilot study, we compared different sequencing-based methods to assess their relative accuracy with respect to distinguishing between viable and non-viable cells, using a live and heat-inactivated model community spiked into bovine milk. The methods used were shotgun metagenomics with and without propidium monoazide (PMA) treatment, RNA-based 16S rRNA sequencing and metatranscriptomics. The results showed that methods were generally accurate, though significant differences were found depending on the library types and sequencing technologies. Different molecular targets were the basis for variations in the results generated using different library types, while differences in the derived composition data from Oxford Nanopore Technologies-and Illumina-based sequencing likely reflect a combination of different sequencing depths, error rates and bioinformatics pipelines. Although PMA was successfully applied in this study, further optimisation is required before it can be applied in a more universal context for complex microbiomes. Overall, these methods show promise and represent another important step towards the ultimate establishment of approaches that can be applied to accurately identify live microorganisms in milk and other food niches.

## Importance

High-throughput DNA sequencing has been useful in characterising microbial communities in various food-related environments. However, there is potential for misleading compositional data to be generated, because of the sequencing of DNA from dead microorganisms. The differentiation of live/dead cell states is also particularly important when making decisions on food safety or spoilage, when DNA from cells recently inactivated through food processing/preservation steps can be problematic. Overcoming this would enhance the power of DNA sequencing for food microbiology testing. In this study, 4 different sequencing-based methods were evaluated for their ability to differentiate between viable and non-viable cells spiked into a milk matrix. We identified differences between data derived by these methods due to differing molecular targets and sequencing technologies used. This study provides insight that will support the enhancement of sequencing-based approaches to better characterise live microbes in food-related environments.

## Introduction

Microorganisms are ubiquitous and their presence and activity influence the niches that they inhabit. The use of high-throughput sequencing methods has expanded the understanding of microbiomes in clinical, food and environmental settings. However, the majority of the commonly used sequencing methods, such as amplicon or shotgun metagenomic sequencing, target (meta)genomic DNA (gDNA) in a manner that does not distinguish between DNA from microorganisms that are viable from those that non-viable microbes (Emerson et al., 2017). This is important as some studies have found that DNA from non-viable cells and other extracellular DNA can persist in various environments, such as water, soil, food and the built environment, for days to weeks (Nocker and Camper, 2006; Li et al., 2017). Not being able to distinguish between viable and non-viable microorganisms can lead to an overestimation of the relative abundance of particular taxa and/or the active metabolic processes that they encode (Carini et al., 2016; Mancabelli et al., 2021). This, in turn, has potentially problematic implications when detecting spoilage or pathogenic microbes, determining bioburdens and the effectiveness of antimicrobial/cleaning treatments or processes (Carini et al., 2016; Emerson et al., 2017). In a food setting, the accurate identification of live pathogenic or spoilage microbes is of considerable importance with respect to decision making processes relating to food safety, quality and product release. Therefore, the future widespread application of high-throughput sequencing as a tool by the food industry will be reliant on the ability to distinguish between live and dead cells in a food or food-related community.

Rapid culture-independent bacterial viability analysis is complex, and viability assays often query several different aspects such as cell membrane integrity, cellular metabolic activity or the presence of functional nucleic acids that allow for transcription/translation and DNA replication (Hammes et al., 2011; Emerson et al., 2017). Traditional culture-based techniques continue to be most extensively used in food microbiology testing and are the industry standard for detecting viable foodborne microorganisms (Foddai and Grant, 2020). However, these methods can only detect a proportion of the viable microorganisms present in a sample as they rely on isolation and growth of a subset of microorganisms on culture media, which is not usually reflective of the entire community and which can result in an underestimation of the levels of specific microorganisms (Gupta et al., 2019; Foddai and Grant, 2020). To address issues associated with culture-based approaches, nucleic acid-based culture-independent methods have been studied and employed to detect viable microorganisms. Cell viability dyes, such as ethidium monoazide (EMA) or propidium monoazide (PMA), bind to accessible DNA that are not protected by a cell membrane after photoactivation and prevents further amplification of any non-intact DNA in downstream steps (Nocker and Camper, 2006; Marotz et al., 2021; Wang et al., 2021). Methods involving the use of such dyes have been used with nucleic acid-based methods such as polymerase chain reaction (PCR), quantitative PCR (qPCR) and amplicon and metagenomic sequencing to detect viable microbes in clinical, environmental and food microbiomes (Carini et al., 2016; Kable et al., 2019; Marotz et al., 2021). Ribonucleic acid (RNA)-based techniques focused on the transcriptome have also been investigated with a view to distinguishing between live and dead cells in samples. As RNA has a shorter half-life than DNA, the use of RNA as a molecular target can more accurately represent the living/viable microbial population (Emerson et al., 2017; Gomez-Silvan et al., 2018). Indeed, the detection of messenger (mRNA) or ribosomal ribonucleic acid (rRNA) has been used to identify metabolically active cells in various samples (Mira Miralles et al., 2019; Yang et al., 2020; Zhou et al., 2020). Metatranscriptomics targets mRNA and provides information relating to the identity of the microorganisms that are metabolically active and the genes that these microbes are expressing (Chen et al., 2017). The more targeted use of 16S rRNA has also been shown to have potential in identifying active bacteria in foods (Mira Miralles et al., 2019).

Here we investigate the relative success with which four sequencing-based methods distinguish between viable and non-viable bacteria in milk samples. A five-strain model community consisting of different numbers and proportions of live and dead cells was spiked into bovine milk samples before undergoing two DNA-based approaches, shotgun sequencing with and without prior PMA treatment, and two RNA-based approaches, metatranscriptomics and RNA-based 16S rRNA sequencing, on both representative Illumina (shorter read length; higher read numbers) and Oxford Nanopore Technologies (ONT; longer read length; lower read numbers).

## Materials and methods

### Model community and samples

Strains employed to subsequently create a model community were obtained from the Teagasc Dairy Production Centre (DPC) culture collection. These strains were *Bacillus velezensis* DPC 3313, *Escherichia coli* DPC 6912, *Lactococcus lactis* DPC 4268, *Pseudomonas proteolytica* DPC 6056 and *Staphylococcus haemolyticus* DPC 5987 and represent bacteria found in the bovine milk microbiome (Quigley et al., 2013). Strains were grown on tryptic soy agar (TSA; Thermo Fisher, Ireland) overnight at 30°C (*B. velezensis* and *P. proteolytica*) or 37°C (*E. coli*, *L. lactis* and S. *haemolyticus*) to generate fresh cultures. Cells were diluted in 10 ml phosphate-buffered saline (PBS; Thermo Fisher) to obtain a bacterial suspension of a density equivalent to approximately 10^7^ cells/mL for each strain. Equal volumes of strains were combined to generate a 5-strain model community for spiking into milk samples. This mixture was used as a live model community and a dead model community was generated through heat inactivation at 95°C for 15 min (resulting in an absence of subsequent growth on TSA).

Store-bought UHT skim milk was used for milk spiking studies in order to ensure a low load of background microorganisms. Two different viability conditions were used for spiking; live (consisting of 1 ml of the live-cell model community) and dead (consisting of 1 ml of the dead-cell model community). Cells were spiked into 10 ml of milk in triplicate and left to incubate at room temperature overnight. Unspiked milk samples and both the live and dead bacterial model communities, which did not undergo any treatment and were used for spiking, were used as controls. Milk samples were then centrifuged at 4,500 × g for 15 min at 4°C, the supernatant was discarded, and the cells pellets were subjected to two washing steps whereby the pellets were resuspended in sterile PBS and centrifuged at 13,000 × g for 1 min, after which the supernatant was discarded. Samples used for Shotgun and PMA-Shotgun metagenomic analysis were resuspended in 1 ml sterile PBS while, for samples used for metatranscriptomic- and 16S rRNA-based analysis, pellets were resuspended in 500 μl RNALater and stored at -80°C before extraction.

### PMA treatment and DNA extraction

Each 1 ml sample was divided into 2 × 500 μl samples, one of which was subjected to a PMA (PMAxx dye, Biotium, CA, United States) treatment and the other without PMA treatment. Preliminary investigations were performed to optimise the final PMA concentration, incubation and light exposure time with the quantification of total bacteria performed using the Femto Bacterial DNA Quantification kit (Zymo Research, CA, USA). The final parameters for PMA treatment involved the use of a final PMA concentration of 20 μM and incubation in the dark at room temperature and light exposure with the PMA-Lite™ LED photolysis device (Biotium, USA) for 30 min. Following PMA treatment, both PMA-treated and PMA-free samples were subjected to DNA extraction using the MolYsis complete5 kit (Molzym GmBH & Co. KG, Bremen, Germany), with 50 μl of DNA eluted for downstream sequencing. The MolYsis kit was used as it had been found to significantly improve microbial sequencing depth for milk samples (Yap et al., 2020). gDNA was quantified using the Qubit dsDNA HS assay kit (Invitrogen) and stored at at -20°C before library preparation. Total bacteria levels were again quantified using the Femto Bacterial DNA Quantification kit (Zymo Research).

### Illumina DNA library preparation and shotgun metagenomic sequencing

A total of 18 samples (6 PMA-treated, 6 non-PMA-treated, 6 controls) were prepared for shotgun metagenomic sequencing according to Illumina Nextera XT library preparation kit guidelines, with the use of unique dual indexes for multiplexing with the Nextera XT index kit (Illumina). Following indexing and clean up, samples were pooled to equimolar concentration of 1 nM. Samples were sequenced on an Illumina NextSeq 500 sequencing platform with a V2 kit, at the Teagasc DNA Sequencing Facility, using standard Illumina sequencing protocols.

### MDA amplification and Oxford Nanopore DNA metagenomic library preparation and sequencing

Whole metagenome amplification was performed on samples using multiple displacement amplification (MDA) with the REPLI-g UltraFast Mini kit (Qiagen, West Sussex, United Kingdom), according to the manufacturer’s instructions. After amplification, DNA was quantified using the Qubit dsDNA BR assay kit (Invitrogen) and normalised to 400 ng in 7.5 μl. Two libraries were prepared, with each library containing 9 samples (6 samples (PMA-treated or non-PMA-treated) and 3 controls) per flowcell. The Rapid Barcoding kit (SQK-RBK004, Oxford Nanopore Technologies, United Kingdom) was used to prepare the libraries. For this, DNA was tagmented and barcodes were attached to fragments before pooling, followed by clean-up using Ampure XP beads (Beckman Coulter) to concentrate the pooled library directly prior to sequencing. DNA was sequenced using a GridION (release 19.12.6) with single flow cells for each library (*R* 9.4.1) that were primed prior to loading libraries with MinKNOW (core 3.6.5) and integrated basecalling by Guppy (3.2.10).

### RNA extraction

Samples to be used for 16S rRNA and metatranscriptomic-based analysis were subjected to RNA extraction using a TRIzol chloroform protocol with on-column DNase purification with the PureLink RNA Mini kit (ThermoFisher Scientific). Briefly, samples suspended in RNALater were first centrifuged at maximum speed (17,000 × g) for 1 min at 4°C, after which the supernatant was discarded. 1 ml of TRIzol reagent was added to each sample and mixed through pipetting up and down to disperse the pellet, followed by incubation for 10 min at 60°C. To each sample, 200 μl of chloroform was added and the tubes were shaken vigorously by hand for 15 s before incubation at room temperature for 3 min. The samples were then centrifuged at 12,000 × g for 15 min at 4°C, during which the mixture separates into a lower, red phenol-chloroform phase, an interphase, and a colourless upper aqueous phase which contains the RNA. Approximately 400 μl of upper aqueous phase of RNA was transferred to a RNase-free tube where an equal volume of ice-cold 100% ethanol was added for precipitation and vortexed for even mixing. The samples were then purified through on-column DNase treatment following the manufacturer’s protocol with final elution at 30 μl in RNase-free water. RNA concentration was quantified using the Qubit RNA HS assay kit (Invitrogen) and quality checked using an Agilent Bioanalyzer with the RNA 6000 pico assay kit (Agilent). RNA was stored at -80°C for use in downstream library preparation steps.

### cDNA synthesis

For 16S methods, 20 μl of RNA for each of the 9 samples (6 samples and 3 controls) were used for cDNA synthesis with the Superscript IV First Strand Synthesis System (Invitrogen) according to the manufacturer’s instructions, using 2 μM of the reverse primer of the *V*3-*V*4 region of the 16S rRNA gene that are commonly used in Illumina 16S sequencing. The cDNA generated was stored at -20°C overnight before use in library preparation.

### Illumina 16S-rRNA library preparation and sequencing

Five microliters of the cDNA generated was used in library preparation according to Illumina 16S Metagenomic Sequencing Library Preparation guidelines, with the use of dual indexes for multiplexing with the Nextera XT index kit (Illumina). Following indexing and clean up, samples were pooled to equimolar concentration of 20 nM. Samples were sequenced on an Illumina MiSeq sequencing platform with a V3 kit, at the Teagasc DNA Sequencing Facility, using Illumina sequencing protocols.

### Oxford Nanopore 16S-rRNA library preparation and sequencing

The cDNA generated was normalised to 10 ng in 10 μl for use in library preparation with the 16S Barcoding kit (SQK-RAB204, Oxford Nanopore Technologies) according to the manufacturer’s instructions. Briefly, amplification of the 16S gene was done using barcodes, followed by library clean up using Ampure XP beads (Beckman Coulter) and pooling to 10 ng/μL. Sequencing adapters were attached before the loading of libraries on a single primed flow cell (R 9.4.1) and sequenced on the GridION (release 19.12.6) with MinKNOW (core 3.6.5) and integrated basecalling by Guppy (3.2.10).

### rRNA depletion

For metatranscriptomics, rRNA depletion was carried out using the QIAseq FastSelect–5S/16S/23S kit (Qiagen) according to the manufacturer’s instructions with 20 μl of each of the 9 samples (6 samples and 3 controls). The first step of RNA fragmentation was performed at 89°C for 7 min. After bead clean up, rRNA-depleted RNA was stored at -80°C before use in library preparation.

### Illumina metatranscriptomics library preparation and sequencing

First strand cDNA synthesis was performed using the NEBNext Ultra II Directional RNA Library Prep Kit (Brennan & Co., Dublin, Ireland) with 10 μl of rRNA-depleted RNA. Library preparation was performed according to the manufacturer’s instructions. Seven cycles were used for PCR amplification during indexing of adapter ligated DNA. The quality and quantity were measured using a high sensitivity DNA chip on the bionanalyzer (Agilent) and Qubit HS dsDNA kit (Invitrogen), respectively. An additional clean up step was done due to the presence of primer and adapter dimers before the quality and quantity was measured. Samples were pooled to equimolar concentration of 4 nM before sequencing on the Illumina NextSeq 500 using a mid-output (2 × 75 bp kit) at the Teagasc DNA Sequencing Facility, using Illumina sequencing protocols.

### Oxford Nanopore metatranscriptomics library preparation and sequencing

rRNA-depleted RNA was normalised to 100 ng in 7.5 μl for use in library preparation with the Direct cDNA Native Barcoding kit (SQK-DCS109, Oxford Nanopore Technologies) with the native barcoding expansion kit (EXP-NBD104, Oxford Nanopore Technologies) according to the manufacturer’s instructions. Briefly, reverse transcription and strand switching was done to prepare full-length cDNAs followed by barcode ligation. Barcoded samples were pooled prior to adapter ligation before a final clean up with Ampure XP beads (Beckman Coulter) before loading the library onto a single primed flow cell (*R* 9.4.1) and sequenced on a GridION (release 19.12.6) with MinKNOW (core 3.6.5) and integrated basecalling by Guppy (3.2.10).

### Bioinformatic analysis

Quality checks and adapter trimming for Illumina methods (shotgun, PMA-shotgun and metatranscriptomics) were done with FastQC (v. 0.11.8; Andrews, 2010) and cutadapt (v. 2.6; Martin, 2011) and host reads were aligned to the bovine genome (*Bos taurus*) and removed with Bowtie2 (v. 2.4.4; (Langmead and Salzberg, 2012). Quality checks for ONT methods (shotgun, PMA-shotgun and metatranscriptomics) were done with MinIONQC (Lanfear et al., 2019) and Nanoplot (v. 1.28.2; De Coster et al., 2018), with adapter trimming done with porechop (v. 0.2.4). Host reads were aligned with Minimap2 (v. 2.17-r974) to the bovine genome (*Bos taurus*) (Li, 2018). Sortmerna (v. 2.1b) was used for rRNA removal prior to the analysis of metatranscriptomic data (Kopylova et al., 2012). Taxonomic classification for all metatranscriptomics, shotgun and PMA-shotgun methods was performed with Kraken2 (2.0.7) using the Genome Taxonomy Database (release 89) which contains Bacteria and Archaea (Parks et al., 2018, 2020, 2022; Wood et al., 2019). For the Illumina 16S rRNA method, quality control, adapter trimming and denoising was done with Qiime2 (2021.2) with cutadapt and DADA2 (Martin, 2011; Callahan et al., 2016; Bolyen et al., 2019). For the ONT 16S rRNA method, analysis was performed using the q2ONT pipeline,^1^ which uses porechop (v. 0.2.4) to demultiplex reads and trimmomatic (0.38) to discard reads shorter than 1,400 bp and crop reads to that length. Qiime2 (2021.2) was used for the remaining steps, where sequences were dereplicated and chimeric sequences were filtered out with vsearch at 85% identity before reads were aligned with mafft and highly variable positions were masked and filtered out. For both 16S rRNA methods, taxonomy was assigned with the Qiime2 trained classifier using the Silva 138 database (Quast et al., 2012).

### Statistical analysis and data visualization

Performance metrics were calculated in *R* (4.1.2; R Core Team, 2015), with the relative abundances of a model community strain correctly (TP) and incorrectly (FP) quantified by the method, along with the relative abundances of other taxa present in the sample correctly (TN) and incorrectly (FN) quantified. The following metrics were used:

1. Accuracy is the correct quantification of model community and other taxa across all observations, A = TP + TN/TP + TN + FP + FN
2. Precision is the fraction of correctly quantified model community strains within abundances of taxa identified as the model community, P = TP/TP + FP
3. Sensitivity is the fraction of correctly quantified model community strains within abundances of the actual model community strains, S = TP/TP + FN
4. F-score is the harmonic mean of precision and sensitivity, F = (2 × P × S)/(P + S)

Diversity analysis was done with the vegan package (Oksanen et al., 2015), with beta diversity calculated as Bray-Curtis metrics, visualised in a principal coordinate analysis plot. The “adonis” function from the vegan package was used to calculate the permutational analysis of variance (PERMANOVA) to determine differences in composition of the community between groups of samples (number of permutations = 999). Hierarchical clustering was done with dendextend (Galili, 2015) using Bray-Curtis distances. Data was cleaned, analysed and visualised in R with ggplot2, tidyverse and ggpubr packages (Wickham, 2011; Wickham et al., 2019; Kassambara, 2020).

## Results

**Figure 1:**
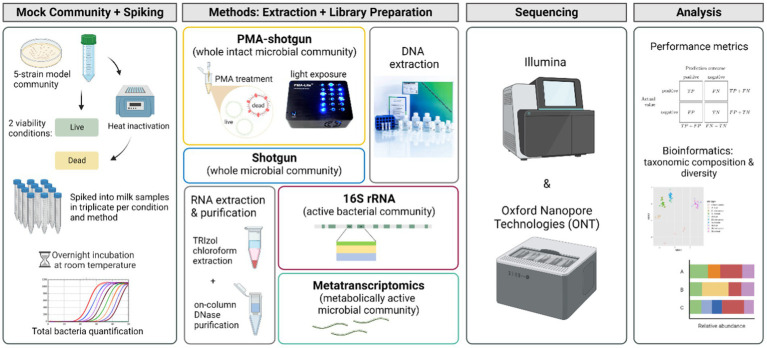
Experimental design of the study (created with BioRender.com).

To determine the relative success with which sequencing-based methods could distinguish live and dead cells in milk, ultra-heat treatment (UHT) bovine milk was spiked with a 5-strain model community of representative bacteria found in milk (*B. velezensis* DPC 3313, *E. coli* DPC 6912, *L. lactis* DPC 428, *P. proteolytica* DPC 6056 and *S. haemolyticus* DPC 5987*)* at two different viability conditions, before undergoing different extraction and library preparation approaches and sequencing. Four methods, shotgun metagenomics with (PMA-shotgun) and without PMA treatment (Shotgun), metatranscriptomics (MetaT) and RNA-based 16S rRNA sequencing (16S rRNA), sequenced on both Illumina and Oxford Nanopore Technologies (ONT) platforms, were evaluated and compared to determine their relative efficacy in differentiating viable and non-viable cells (Figure 1). Sequencing depth differed depending on the platform and the application (Supplementary Table S1). We did not endeavour to sequence at matching depths as the focus was applying the technologies at the depth corresponding to those at which the respective platforms are typically used.

### Taxonomic classification of model community strains was accurate to genus level across all methods

In general, the model community taxonomy was determined with one classifier to ensure consistency across methods. This classifier, Kraken2, was previously found to efficiently characterise the milk microbiome when compared to other classifiers (Yap et al., 2020). In addition, a trained classifier in Qiime2 was used for the analysis of 16S rRNA data (Illumina-sequenced 16S rRNA: i-16 s and ONT-sequenced 16S rRNA: o-16 s). Comparing across methods, the taxonomic identity of the model community was consistent to genus level in all cases (Table 1). This was also the case at species level, where assignment at species level was possible, with the exception that reads generated from Illumina sequencing were assigned as *Staphylococcus hominis*, while the same model community strain was classified as a *Staphylococcus haemolyticus* when ONT data was analysed. However, this is not unexpected as S. *hominis* is classified under the S. *haemolyticus* group of bacteria (Becker et al., 2014). ONT-generated metatranscriptomic data and the short read length 16S rRNA data (regardless of sequencing platform) could not be resolved beyond genus level. Indeed, *Staphylococcus* was not detected from within the ONT-derived metatranscriptomic data, most likely due to insufficient sequencing depth combined with low levels of gene expression by the strain (Supplementary Table S1). For downstream analysis, genus level data was used.

**Table 1:** The highest resolution of taxonomic identity identified of the model community strains across all methods.

Model community strain	Shotgun		PMA-shotgun		Metatranscriptomics		16S rRNA	
	Illumina	Nanopore	Illumina	Nanopore	Illumina	Nanopore	Illumina	Nanopore
*Bacillus velezensis* DPC 3313	*Bacillus velezensis*	*Bacillus velezensis*	*Bacillus velezensis*	*Bacillus velezensis*	*Bacillus velezensis*	*Bacillus*	*Bacillus*	*Bacillus*
*Escherichia coli* DPC 6912	*Escherichia coli*	*Escherichia coli*	*Escherichia coli*	*Escherichia coli*	*Escherichia coli*	*Escherichia*	*Escherichia-Shigella*	*Escherichia-Shigella*
*Lactococcus lactis* DPC 4268	*Lactococcus lactis*	*Lactococcus lactis*	*Lactococcus lactis*	*Lactococcus lactis*	*Lactococcus lactis*	*Lactococcus*	*Lactococcus*	*Lactococcus*
*Pseudomonas proteolytica* DPC 6056	*Pseudomonas_E proteolytica*	*Pseudomonas_E proteolytica*	*Pseudomonas_E proteolytica*	*Pseudomonas_E proteolytica*	*Pseudomonas_E proteolytica*	*Pseudomonas_E*	*Pseudomonas*	*Pseudomonas*
*Staphylococcus haemolyticus* DPC 6283	*Staphylococcus hominis*	*Staphylococcus haemolyticus*	*Staphylococcus hominis*	*Staphylococcus haemolyticus*	*Staphylococcus hominis*	*−*	*Staphylococcus*	*Staphylococcus*

Total bacteria qPCR was performed to quantify the bacteria in each sample and the log number of cells/mL of each microorganism per sample was estimated using a combination of qPCR results and relative abundance values. The abundances of cells recovered from Live samples were greater than those in the heat-treated samples (Supplementary Figure S1). Some reads were classified into genera other than those corresponding to the 5 spiked strains (Supplementary Table S2), and some of these were also detected in the unspiked milk samples (Supplementary Table S3). The reads classified as others were not prioritised in the further analysis due to the particular focus on differentiating between the members of the model community in the Live and Dead samples.

### Methods were accurate though the precision and sensitivity varied

**Figure 2:**
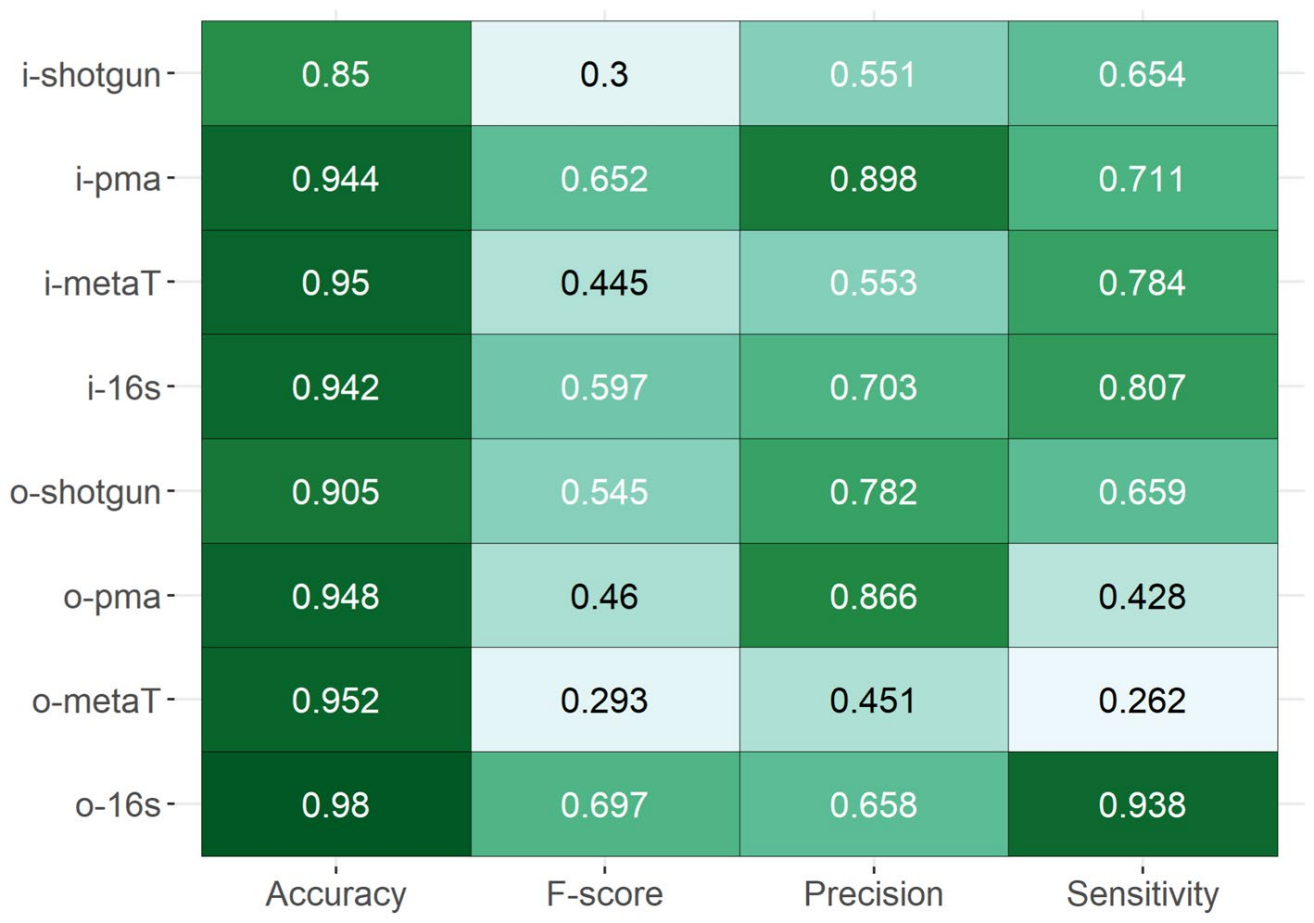
Overall performance of methods based on the accuracy, F-score, precision, and sensitivity.

To evaluate the performance of each method, several performance metrics such as accuracy, precision, sensitivity and F-score were calculated. The comparisons were made between the abundances of the live and dead model community controls that had been sequenced and analysed with the various methods. Overall, most approaches showed a high level of accuracy, which is the fraction of correctly identified taxa (model community or other) relative to all observations, with a median of 0.939 ± 0.046 (Figure 2). However, the F-score, which gives a sense of how precise and sensitive the method is, revealed that only half of the methods scored at least 0.5, indicating issues in precision or sensitivity in these instances. The two PMA-shotgun sequencing and analysis methods yielded outputs that were most precise, while the 16S rRNA methods were the most sensitive, including when separated into both live and dead spiked samples (Figure 2, Supplementary Figure S2). ONT-sequenced 16S rRNA (o-16 s) was the most accurate and had the best overall F-score, while metatranscriptomics (i-metaT) and PMA-shotgun (i-pma) had the highest accuracy and F-score out of all the Illumina-based methods, respectively.

### Significant differences were observed between data depending on library types (DNA and RNA) and sequencing methods (Illumina and ONT)

**Figure 3:**
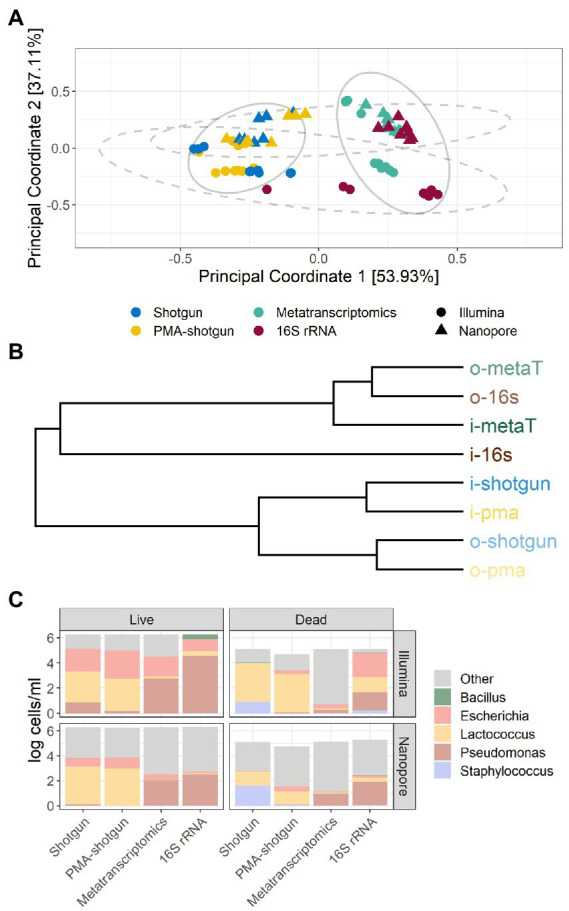
Beta diversity and taxonomic profiles of samples. **(A)** Bray-Curtis principal-coordinate analysis (PCoA) plot, with ellipses representing clustering by DNA- and RNA-based methods (solid) and sequencing method (dashed) and **(B)** hierarchical clustering of methods based on Bray-Curtis distances. **(C)** Composition of taxa in samples based on live or dead spiked model community, by the evaluated methods and sequencing method.

Beta diversity analysis was employed to determine the differences between the outputs generated from the different approaches taken, and several sample clusters were apparent (Figure 3A). Samples first clustered by library type, whereby the outputs from DNA-based analysis (Shotgun and PMA-shotgun) were significantly distinct from those generated using the RNA-based approaches (metatranscriptomics and 16S rRNA; PERMANOVA, *R*^2^ = 0.457, *p* < 0.01), and also by sequencing methods, with Illumina-sequenced and ONT-sequenced outputs clustering apart (PERMANOVA, *R*^2^ = 0.195, *p* < 0.01). Differences in nucleic acid input and sequencing methods used were also apparent in the hierarchical clustering of the Bray-Curtis distances (Figure 3B). The composition of taxa varied across library types, with *Escherichia* and *Lactococcus* being found at greater abundance when DNA-based methods were used, while *Pseudomonas* was the most active on the basis of RNA-based methods (Figure 3C). In particular, the Illumina-sequenced 16S rRNA data points clustered away from those generated using other methods. This was also obvious in the taxonomic composition where abundances of other taxa besides the model community was lowest in i-16S compared to other methods (Figure 3C).

**Figure 4:**
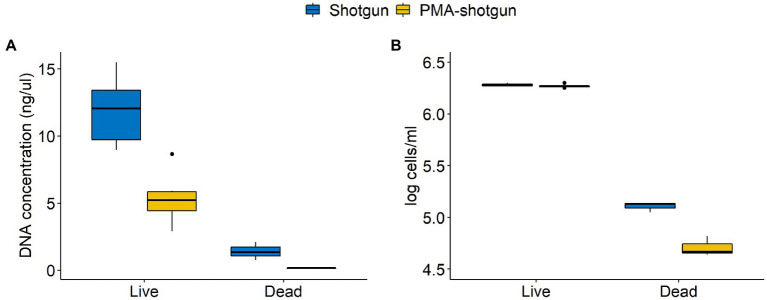
Differences in concentrations of **(A)** DNA quantified using Qubit and **(B)** cells quantified through qPCR between Shotgun and PMA-shotgun methods for live and dead spiked samples.

Among the DNA-generated dataset, the effect of PMA was clear, with lower DNA and overall calculated cell counts observed for samples that underwent PMA treatment (Figure 4). Notably, although the Shotgun methods do not exclusively select for viable cells, the accuracy of those methods were comparable to the PMA-shotgun methods (Figure 2). Precision was higher in PMA-shotgun methods compared to the Shotgun methods, while sensitivity was higher for i-pma and lower for o-pma when compared to i-shotgun and o-shotgun, respectively. In terms of the composition, most of the abundances of model community taxa in PMA-shotgun samples were lower in total cells/mL than those in Shotgun samples, apart from *Escherichia* cells/mL, which was higher in PMA-shotgun methods than Shotgun for both live and dead spiked samples (Figure 3C). The cell counts of *Bacillus* and *Pseudomonas* were generally lower, with the exception of i-shotgun where both taxa were found in abundances greater than the other DNA-based methods. *Lactococcus* was the most abundant of the model community taxa quantified, which was consistent across the two methods, with exception of o-shotgun, which detected higher abundances of *Staphylococcus*. *Staphylococcus* was clearly detected in the Shotgun samples spiked with the dead cells whereas levels were negligible in the PMA-shotgun samples. Between sequencing methods, distinct separation was found between Illumina and ONT methods (Figures 3A,B) and, in terms of composition, ONT methods detected higher abundances of other non-model community taxa, compared to the Illumina methods (Figure 3C).

Although there were no obvious differences in the accuracy of the methods across the different RNA-based approaches, the 16S rRNA method outperformed the metatranscriptomics approaches with respect to precision and sensitivity (Figure 2). In general, methods were more sensitive than precise, except for o-metaT which had poor sensitivity. *Staphylococcus* reads were detected in low abundances across all RNA-based methods, while read numbers corresponding to *Pseudomonas* were consistently found at high abundance (Figure 3C). Similar to the DNA-based methods, clear separation between library types was seen between Illumina- and ONT-based methods (Figure 3). As mentioned above, Illumina-sequenced 16S rRNA was the most distinct, clustering separately from other methods and with the lowest proportion of other taxa identified compared to the other RNA-based methods (Figures 3A,B). It was the only method that had detectable levels of *Bacillus* in live samples and its samples spiked with the dead-cell model community had a taxonomic profile that differed from the other samples spiked with dead cell concentrations (Figure 3C).

## Discussion

While DNA sequencing-based approaches have enabled the characterisation of complex microbial communities more efficiently, they generally provide information that reflects all microbial DNA present in the community and do not specifically select for viable microorganisms (Carini et al., 2016; Wang et al., 2021). In various settings, the ability to distinguish between viable or metabolically active and non-viable or dead microorganisms can be important and, indeed, necessary in terms of food safety and public health, and for food processing involving live microorganisms. While culture-based approaches are commonly used by industry and public health authorities to determine viability of individual microbes (Emerson et al., 2017), high-throughput sequencing-based methods have great potential as a means of characterising microbial communities in foods or food environments. In this study, we explored 4 sequencing-based approaches to assess the relative success with which they distinguish between live and dead cells of a 5-strain model community in a milk matrix.

All methods performed accurately, despite the slight compositional differences between the two library types. The cause of the difference could possibly relate to the approach of each library type, with the use of PMA or other intercalating dyes taking advantage of cell integrity to remove cells with a compromised membrane (Emerson et al., 2017; Wang et al., 2021), while RNA-based methods examine the activity of cells, as RNA has a shorter average half-life than DNA (Emerson et al., 2017; Li et al., 2017; Gomez-Silvan et al., 2018; Yang et al., 2020). This implies that *Pseudomonas* were most active, while *Escherichia* and *Lactococcus* had the greatest abundance of intact cells. The different targets and the different library preparation methods (amplicon, shotgun and metatranscriptomics) make it difficult to directly compare the methods with each other. Microbial community studies with both types of libraries (DNA and RNA) often perform a single molecular method on the samples, such as 16S rRNA sequencing or qPCR, to allow for comparison of results from DNA and RNA libraries. In these studies, consistent outcomes have been reported in that distinctions have been found between library types in water, sediment, and food samples when both DNA and RNA were extracted and sequenced from the same samples (Ferrocino et al., 2016; Li et al., 2017; Mira Miralles et al., 2019; Pearman et al., 2021).

Our data also reveal clear differences between the outputs of Illumina- and ONT-based sequencing technologies, with samples clustering distinctly based on sequencing technologies used. Differences between sequencing technologies have also been reported in intestinal and nasal microbiota samples with species level classification and the clustering of the microbiome showing significant effects based on the sequencing technology utilised (Heikema et al., 2020; Alili et al., 2021). Besides the known higher error rate of ONT sequencing compared to Illumina (Alili et al., 2021), a few other possibilities could account for this. First, in order to maintain consistency during analysis, bioinformatic tools that are more commonly applied to short read data were used for all the sequencing data generated and, thus, specific tools developed for long read sequencing data analysis were not used. It is possible that improved classification could be achieved should the relevant tools for error correction for long reads be applied to the ONT sequencing data. Another contributory factor is the difference in sequencing depth. ONT sequencing had been previously found to produce a high proportion of unclassified reads when a shallow sequencing depth was employed, and greater sequencing depth (30–50X) has been known to enable self-correction of the error rate that is associated with Nanopore sequencing data (Alili et al., 2021; Delahaye and Nicolas, 2021). These factors could account for the numerical difference in species level classification of the *Staphylococcus* strain, where Illumina sequencing afforded greater taxonomic resolution than ONT sequencing, as *S. hominis* is specifically classified within the *S. haemolyticus* group (Becker et al., 2014). Additionally, for shotgun sequencing with ONT, MDA amplification was needed to generate sufficient quantities of DNA required for sequencing, which has been previously found to introduce some biases (McHugh et al., 2021). Despite all these points, as improvements are made to the sequencing technology and analytical tools, coupled with its advantages in portability, time effectiveness and the possibility of real-time analysis, there is great potential for the use of ONT sequencing in characterising microbial communities in food and food-related environments.

PMA is a photoreactive DNA-binding dye that binds to non-viable cells that have compromised membranes and inhibits further amplification by PCR (Nocker and Camper, 2006). Our results show evidence of the effect of PMA, with more precise results and lower abundances of live and dead cells achieved when compared to regular shotgun sequencing. The use of PMA did not bias the community structure, which adds to their promising application. However, studies have found that the effectiveness of PMA varies significantly (Mancabelli et al., 2021; Wang et al., 2021). Biological matrix, sample biomass, microbial community diversity and experimental conditions have been known to contribute to this variation (Li et al., 2017; Mancabelli et al., 2021; Shen et al., 2021; Wang et al., 2021). The optimisation of dye concentration, incubation and light exposure times are required depending on the samples, which prevents the application of a universal PMA treatment for all types of samples. Moreover, methods based on membrane integrity may result in an overestimation of viable cells as the lethal stress may not lead to the immediate disruption of the cell membrane and dyes used may be ineffective against cells with a hardy cell wall, such as spores (Emerson et al., 2017). Although the close clustering of PMA-shotgun to Shotgun samples could suggest the insufficient or unsuccessful PMA treatment, the low diversity samples created by spiking the simple model community into milk samples could be the reason for such results. Regardless, this further emphasises the need to optimise PMA treatment, which could differ between samples. Therefore, though promising, more work is needed to calibrate PMA concentration and incubation and light exposure times for specific sample matrices before it can be effectively applied to DNA sequencing experiments with confidence.

Within the data generated by RNA-based methods, results differed between metatranscriptomics and 16S rRNA sequencing, which is not surprising as they use different types of RNA as targets. Metatranscriptomics targets mRNA, which has a very short half-life, while 16S rRNA sequencing uses rRNA, which is generally more stable than mRNA and is more abundant in cells (Emerson et al., 2017). In spite of these differences, both mRNA and rRNA have been found to be good markers of bacterial viability, with several studies suggesting that rRNA might provide more accurate taxonomic profiling and may be more successful for low-biomass samples compared to mRNA approaches (Emerson et al., 2017; Yang et al., 2020). Additionally, the cells in both model communities may not have the same physiological state after overnight incubation, which could affect their metabolic activity and the resulting differences between the two RNA-targetting methods. Additionally, cDNA conversion and the different library preparation steps could have introduced biases that could contribute to the differences (Li et al., 2017; Sessegolo et al., 2019). The targeted nature of 16S rRNA sequencing is possibly the reason that the Illumina-sequenced 16S method is the most sensitive and distinct from all the methods (Figure 3). As amplicon sequencing is more widely established and applied, analysis tools for such data are well refined compared to other community-based methods like metatranscriptomics. In spite of this, several studies have employed both RNA- and DNA-based 16S rRNA sequencing concurrently to give a more comprehensive view of both the overall and metabolically active community, which is useful in understanding microbial community dynamics (Gomez-Silvan et al., 2018; Mira Miralles et al., 2019). However, from a practical perspective, RNA-based methods are generally more laborious than DNA-based methods, as RNA is more complicated to handle, which can result in RNA losses during processing (Emerson et al., 2017; Li et al., 2017; Marcelino et al., 2019). In addition to the need for a greater sequencing depth, the metatranscriptomics methods require more processing that could introduce additional biases or losses, which possibly impacted the accuracy of results in our study. Therefore, metatranscriptomics, for now, might not be the most suitable method if the aim is to determine viability alone. It, however, is able to provide useful information on the active microbes and expressed genes that would expand the understanding of microbial community dynamics of foods, food processes or environments (Chen et al., 2017).

Though this study provided insight into sequencing-based methods that could be employed to distinguish between viable and non-viable microbial communities, there were some limitations. First, overnight incubation caused the growth of the model community to levels beyond the expected equal concentrations, causing greater difficulties in determining the success of methods and hindering the analysis of the potential of each method in quantifying live and dead cells. While the kits used were based on prior in-house experience, simultaneous DNA and RNA extractions could have been performed, which could have standardised the laboratory workflow. The use of MDA for ONT DNA-based samples was necessary to obtain sufficient DNA yields for sequencing but may be a confounding factor in the analysis. Lastly, DNA-based 16S rRNA sequencing could have been done to allow for better comparison with the RNA-based method and to help draw further conclusions of the differences between library types.

Despite these challenges, this study gives insight into the use of sequencing-based methods in distinguishing viable and non-viable cells in food samples. While it was difficult to make direct comparisons between the methods due to the differences in their molecular targets, this study shows that differences exist between library types and sequencing technologies, which serves as a stepping-stone to refining these methods. Besides the need to optimise PMA treatments, more complex matrices and more complex microbial communities can be used to test the performance of these methods since the milk samples used had a low microbial density and diversity. Thus, further research is warranted before using these methods to characterise viable microbial communities in food and food-related environments.

## Data availability statement

The datasets presented in this study can be found in online repositories. The names of the repository/repositories and accession number(s) can be found at: https://www.ebi.ac.uk/ena, PRJEB53378.

## Author contributions

MY: conceptualization, methodology, investigation, data curation, analysis, visualization, and writing—original draft and editing. PC: conceptualization, supervision, writing—reviewing and editing, and funding acquisition. OO’S and PO’T: supervision and writing–reviewing. All authors contributed to the article and approved the submitted version.

## Funding

Research was conducted with the financial support of the Irish Dairy Levy.

## Conflict of interest

The authors declare that the research was conducted in the absence of any commercial or financial relationships that could be construed as a potential conflict of interest.

## Publisher’s note

All claims expressed in this article are solely those of the authors and do not necessarily represent those of their affiliated organizations, or those of the publisher, the editors and the reviewers. Any product that may be evaluated in this article, or claim that may be made by its manufacturer, is not guaranteed or endorsed by the publisher.

## Footnotes

^1^ https://github.com/DeniRibicic/q2ONT
