# Supplementary material for: Development of sequencing-based methodologies to distinguish viable from non-viable cells in a bovine milk matrix: A pilot study
Source: Front Microbiol. 2022 Nov 17;13:1036643. doi: 10.3389/fmicb.2022.1036643 (PMC9713316; doi:10.3389/fmicb.2022.1036643)
Supplement: Supplementary file 1 [file Data_Sheet_1.pdf]

SUPPLEMENTAL MATERIAL

TABLE S1. Sequencing quality metrics for each method.

Sequencing quality metrics	Shotgun		PMA-shotgun		Metatranscriptomics		16S rRNA	
	Illumina	Nanopore	Illumina	Nanopore	Illumina	Nanopore	Illumina	Nanopore
**Total reads (post QC)**	51,942,599	195,025	54,999,513	240,167	315,713,655	12,817	861,528	5,109,250
**Total bases**	8,399,308,260	414,568,905	8,817,312,962	529,322,712	20,184,088,333	6,041,520	1,003,649,400	6,555,619,361
**Average reads per sample**	5,771,400	16,252	6,111,057	20,014	35,079,295	1068	95,725	425,771
**Median reads per sample**	5,207,867 ± 1,973,157	16,679 ± 5,370	5,626,993 ± 5,458,311	25,266 ± 14,702	50,532,158 ± 26,316,834	667 ± 1246	105,232 ± 29,638	316,600 ± 521,012
**Median read length**	150	1107	150	910	75	378	250	1111

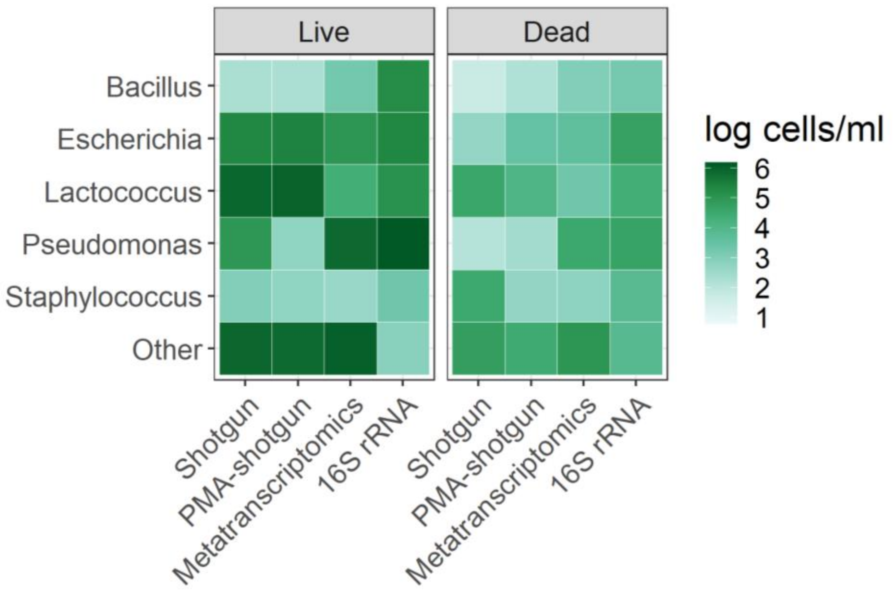

FIG S1. Abundances of samples spiked into Live and Dead conditions, quantified using qPCR and relative abundance data, stratified into the 4 different methods studied.

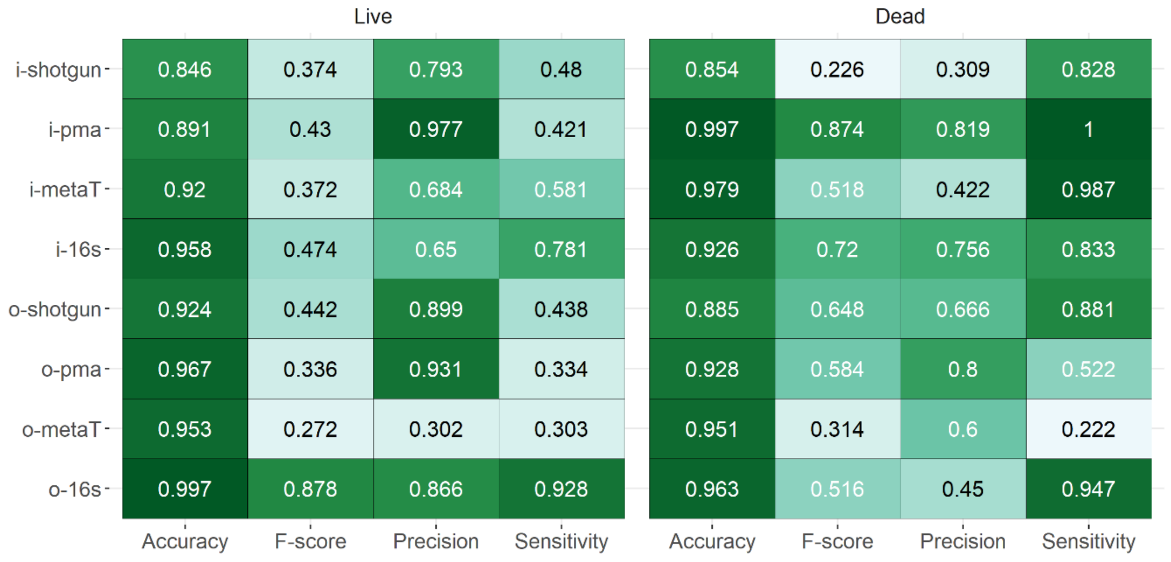

FIG S2. Performance metrics of samples based on comparison of abundances with the live and dead model community controls.

TABLE S2. Other taxa found in spiked milk samples at relative abundances above 0.1%.

	**Shotgun**	**PMA-Shotgun**	**Metatranscriptomics**	**16S rRNA**
**Illumina**	*Streptococcus*	*Streptococcus*	*Alloscardovia*	*g__Thermus*
	Anoxybacillus	*Kocuria*	*Thermus*	*g__Chroococcidiopsis_SAG_2023*
	Thermus	*Romboutsia*	*Acinetobacter*	*g__Anoxybacillus*
	Rothia	*Rothia*	*Anoxybacillus*	*g__Streptococcus*
	Kocuria	*Enterococcus_G*	*Cutibacterium*	*g__Enhydrobacter*
	Microbacterium	*Corynebacterium*	*Streptococcus*	*g__Cloacibacterium*
	Corynebacterium		*Bradyrhizobium*	*g__Leptolyngbya_Es-Yyy1000*
	Bifidobacterium		*Cloacibacterium*	*g__Acinetobacter*
	Leuconostoc		*UBA9464*	*g__Leptolyngbya_PCC-6306*
	CAG-791		*Bacillus_A*	*g__Anaerobacillus*
	Carnobacterium		*Stenotrophomonas*	*g__Kocuria*
	Mogibacterium		*Ralstonia*	*g__Lactobacillus*
	Enterococcus_G		*Kocuria*	*g__Burkholderia-Caballeronia-Paraburkholderia*
	Anaplasma		*Delftia*	
	RUG420		*Lactobacillus*	
	Lactobacillus_C		*Sphingomonas*	
	Romboutsia		*Paenibacillus*	
	Murimonas		*Vibrio*	
	Parageobacillus		*Methylobacterium*	
			Blastomonas	
			Psychrobacter	
			Brevundimonas	
			Sphingobacterium	
			Streptomyces	
			Enterobacter	
			Corynebacterium	
			Mycobacterium	
			Acidovorax_D	
			Moraxella_A	
			Ochrobactrum	
			Herbaspirillum	
			Pseudoalteromonas	
			Chryseobacterium_B	
			Sphingobium	
			Achromobacter	
			Chlamydia	
			Dietzia	

			Paeniglutamicibacter<br>*Microbacterium*<br>*Agrobacterium*<br>*Diaphorobacter*<br>*Kluyvera*<br>*Enterococcus_B*	
**Oxford Nanopore Technologies**	*Streptococcus*<br>*Anoxybacillus*<br>*Thermus*<br>*Clostridium_F*<br>*Parageobacillus*<br>*Acinetobacter*<br>*Hafnia*<br>*Leuconostoc*<br>*Stenotrophomonas*<br>*Rahnella*<br>*Turicibacter*<br>*Yersinia*	*Streptococcus*<br>*Photobacterium*	*Finegoldia*	*d__Bacteria*<br>*p__Proteobacteria*<br>*f__Enterobacteriaceae*<br>*c__Alphaproteobacteria*<br>*g__Acinetobacter*<br>*c__Gammaproteobacteria*<br>*o__Rickettsiales*<br>*g__Enhydrobacter*

TABLE S3. Taxa found in control milk samples at relative abundances above 0.1%.

	**Shotgun**	**Metatranscriptomics**	**16S rRNA**
**Illumina**	*Bacillus*	*Escherichia*	*g__Pseudomonas*
	Enterococcus_G	*Lactococcus*	*g__Escherichia-Shigella*
	Escherichia	*Pseudomonas_E*	*g__Lactococcus*
	Lactococcus	*Staphylococcus*	*g__Bacillus*
	Pseudomonas_E	*Streptococcus*	*g__Staphylococcus*
	Staphylococcus	*Acidovorax_D*	*g__Thermus*
	Streptococcus	*Acinetobacter*	*g__Anoxybacillus*
		Agrobacterium	*g__Enhydrobacter*
		Alloscardovia	*g__Cloacibacterium*
		Anoxybacillus	*g__Streptococcus*
		Blastomonas	*g__Acinetobacter*
		Bradyrhizobium	*g__Lactobacillus*
		Brevundimonas	*g__Leptolyngbya_Es-Yyy1000*
		Chryseobacterium_B	*g__Kocuria*
		Cloacibacterium	*g__Burkholderia-Caballeronia-*
		Corynebacterium	*Paraburkholderia*
		Cutibacterium	
		Delftia	
		Diaphorobacter	
		Kluyvera	
		Lactobacillus	
		Microbacterium	
		Moraxella_A	
		Ochrobactrum	
		Ralstonia	
		Sphingobacterium	
		Sphingobium	
		Sphingomonas	
		Stenotrophomonas	
		Streptomyces	
		Thermus	
**Oxford**	*Acinetobacter*	*Escherichia*	*g__Pseudomonas*
**Nanopore**	*Anoxybacillus*	*Pseudomonas_E*	*d__Bacteria*
**Technologies**	*Clostridium_F*		*p__Proteobacteria*
	Escherichia		*f__Enterobacteriaceae*
	Lactococcus		*g__Escherichia-Shigella*
	Pseudomonas_E		*c__Alphaproteobacteria*
	Rahnella		*c__Gammaproteobacteria*

	Staphylococcus	*g__Bacillus*
	Streptococcus	
	Thermus	
	Turicibacter	
	Yersinia	
	Hafnia	
	Leuconostoc	
	Parageobacillus	
	Stenotrophomonas	
